# Assessment of Children’s Metal Exposure via Hand Wipe, Outdoor Soil and Indoor Dust and Their Associations with Blood Biomarkers

**DOI:** 10.3390/ijerph192114614

**Published:** 2022-11-07

**Authors:** Beibei Wang, Fei Gao, Yujie Li, Chunye Lin, Hongguang Cheng, Xiaoli Duan

**Affiliations:** 1 School of Energy and Environmental Engineering, University of Science and Technology Beijing, Beijing 100083, China; 2 State Key Joint Laboratory of Environmental Simulation and Pollution Control, School of Environment, Beijing Normal University, Beijing 100875, China

**Keywords:** metals, children, hand wipe, outdoor soil, indoor dust

## Abstract

The soil environment contributes considerably to human exposure to metals. This study aimed to comprehensively compare children’s exposure to soil metals using different sampling approaches (i.e., hand wipe, indoor dust and outdoor soil) and assessment strategies, combing the method of external exposure evaluation and the correlation with internal biomarkers. Environmental exposure samples (hand wipe, outdoor soil and indoor dust), blood samples and child-specific exposure factors were simultaneously collected for 60 children aged 3 to 12 years from an area of northwestern China. Eight typical toxic metals were analyzed. Results showed that metal levels in hand wipes were associated with children’s age, years of residency and the ground types of the play areas. Hand-to-mouth contact was an important pathway for children’s metal exposure, with the corresponding oral exposure cancer risk to Cr already exceeding the maximum acceptable level. In comparison, metal concentrations in hand wipes were one to seven times higher than those in outdoor soil and indoor dust. Even greater discrepancies were found for the estimated exposure dose, which could lead to differences of several to dozens of times. In addition, Pb, Mn and Cr in hand wipes were significantly correlated with those in blood, whereas no relationships were found with soil and dust. This study indicates that the selection of different sampling and assessing strategies could lead to great differences in children metal exposure outcomes. It also suggests that hand wipe, which could reflect the true and integrated exposure level and the individual difference, serves as a better matrix to assess children’s metal exposure compared to soil and dust. Further studies should standardize the sampling method for hand wipes and verify its applicability for other age groups.

## 1. Introduction

With the presence originating from both natural sources and anthropogenic activities involving fossil fuel burning, vehicle exhaust and industrial activities, toxic metals are widespread in the environment and attract great attention [1,2]. As the pollutants sink, soil is an important matrix of human exposure to toxic metals. Toxic metal exposure is associated with a range of health outcomes in children. For instance, arsenic exposure was associated with the impairment of cognitive function, kidney disorder, skin cancer, lung cancers and renal cancer [3,4,5]. Epidemiological studies found that lead exposure was associated with neurodevelopmental toxicity, immune-mediated respiratory disease, intellectual disability and learning deficits in children [6,7]. The evidence of a threshold for lead-induced effects has not been found [8]. Long-term, low-dose cadmium exposure could result in bone damage, renal injury and nervous system outcomes [9,10,11]. Although zinc and copper are necessary elements in the human body, excessive exposure could lead to the impairment of important metabolic pathways [12].

The soil environment contributes considerably to human exposure to pollutants. When assessing the health risk of soil exposure, the concentration of contaminants in soil or dust samples collected from a single location of an exposed participant, such as soil from the residential courtyard and dust from the living room, were generally utilized to evaluate the entire exposure level [13,14]. However, children may spend their time in other places, such as in vehicles, in school or in public entertainment occupancies, and thus sampling in a single environment could not represent the comprehensive exposure level from multiple environments. In addition, different sample-sieving strategies were applied. For instance, some studies sieved the soil or dust to 2 mm [15], some sieved to 250 μm [16], some sieved to 149 μm [17] and others even sieved to 120 μm [18]. It is worth noting that pollutant concentrations differed greatly among soil particle fractions and it is still unclear the exact particle size of the human-ingested and dermal-contacted soil/dust [19]. Therefore, the inconsistence in sample-sieving methods might facilitate the bias in assessment results and the incomparability across studies. 

In comparison with traditional soil and dust samples, hand wipes, an emerging matrix, have been demonstrated to be a useful tool to evaluate the exposure to several semi-volatile organic compounds (SVOCs) in microenvironments [20,21]. In addition, previous studies showed that contaminants such as FRs and PFASs in hand wipes exhibited significant correlations with those in biological samples [22,23]. It was found that hand-dermal absorption was an important exposure pathway for SVOCs, while oral ingestion was the prominent pathway for human exposure to toxic soil metals, with an approximately 90% contribution deriving from hand-to-mouth contact [24,25,26]. Therefore, there are still knowledge gaps in understanding whether the hand wipe is a more suitable matrix for estimating the toxic metal exposure in contrast to traditional soil or dust methods and its influence on exposure risk using different sampling and assessment strategies. Moreover, to the best of our knowledge, little is known about the metal levels in hand wipes and evaluating the corresponding exposure risk through hand-to-mouth and dermal contacts. 

To address these gaps, studies on a comprehensive comparison of the sampling and assessing campaigns combing differences in external exposure and the association with internal exposure are needed. The objectives of this study were: (1) to investigate and evaluate the metal exposure in hand wipes via hand-to-mouth and dermal contact; (2) to compare the metal exposure using difference sampling and assessing methods (hand wipes vs. indoor dust vs. outdoor soil); and (3) to explore the correlation between external exposure levels and internal exposure to metals. 

## 2. Materials and Methods

### 2.1. Study Design

A total of 60 children from an area of northwestern China, with the number being evenly distributed in two age groups (2–6 years and 7–12 years), were randomly recruited to participate in the current study. Industries featuring new materials, chemical recycling, biological medicine and coal storage and transportation were the main economic resources of the local area. It was observed that food children consumed was not locally grown, with the metal levels relatively low, as determined in previous studies [27]. Thus, we assumed that soil was the key exposure pathway for the local children. The blood sample, hand wipe, indoor dust from the living place and outdoor soil from the outside place where children spent most of their time were collected simultaneously for each child in September 2020. Participants were also required to fill in a short questionnaire with the help of their guardians through a face-to-face interview to obtain their basic information and potential exposure influencing factors. This study was approved by the Ethics Review Committee of USTB (University of Science and Technology Beijing). Written informed consent forms were obtained from the participants and their guardians before conducting the sampling and survey.

### 2.2. Sample Collection

Hand wipe collection: Hand wipe samples were collected using gauze pads (7.5 × 7.5 cm). After being balanced at a constant temperature and humidity for 48 h in an ultra-clean laboratory, each pad was weighed to get a pre-sample weight. One pad was used for each child, who was asked to keep their hands unwashed at least 1 h before sampling. During sampling, the pad was first immersed in 3 mL deionized water and then was applied to wipe the entire surface of children’s hands from wrist to fingernails. After being freeze dried and balanced at a constant temperature and humidity for 48 h in the ultra-clean laboratory, each pad was weighed again to get a post-sample weight. Thus, the amount of dust adhering to each child’s hands could be obtained by the weight difference of the pre- and post-samples. The weighed pad was stored separately in a polypropylene bag and kept at −20 °C.

Outdoor soil collection: Soil samples were collected from the outside place where children spent most of the time during the sampling period through scraping the top layer of soil (0–2 cm) from an area of 100 cm^2^. All soil samples were sieved through a 0.25 mm mesh, which was considered to be the particle size humans are most likely to be exposed to [28].

Indoor dust collection: Indoor dust samples were collected from the floors of children’s living place using a dust-free brush. Each sample consisted of 4 to 5 sub-samples mixed evenly. All dust samples were also sieved through a 0.25 mm mesh. 

Blood collection: A sample of 3 mL of venous blood was collected in a vacutainer tube containing sodium heparin anticoagulant from each participant by the local professional nurse. All blood samples were stored at −20 °C until pre-processing.

Questionnaire survey: The survey was employed through the face-to-face interview. The questions covered (1) the demographic information about the participants, including age, gender and educational level; (2) the exposure factors used in the exposure assessment model, involving body weight, hand-to-mouth contact frequency and hand-to-dust/soil contact frequency; (3) personal behavior, such as hand-washing frequency and the frequency of playing with soil; and (4) living conditions, including house type, ground type of the play area and years of residency. 

### 2.3. Sample Pretreatment and Analysis

Hand wipes: Pads were digested in an acid-cleaned Teflon vessel with 12 mL concentrated HNO_3_ and 2 mL HF using a microwave digestion system (CEM, MARS-5, North Carolina, USA). The digestion procedure was as follows: the temperature gradually rose to 120 °C in 600 s and was maintained for 600 s, then rose to 160 °C in 480 s and was maintained for 900 s and finally rose to 180 °C in 480 s and was maintained for 1500 s. The digestion residue was transferred to the polytetrafluoroethylene tube and digested again at 100 °C using an electric heating panel for 4 h. Finally, the solution was diluted to 25 mL with deionized water, filtered through 0.45 μm Teflon filter and stored at −20 °C until analysis. 

Soil and dust: A 0.25 g soil or dust sample was placed in the Teflon vessel with 6 mL HNO_3_, 3 mL HCL and 2 mL HF. The digestion process was the same as the hand wipe samples. 

Blood: After being shaken well, 1 mL whole blood was placed in the Teflon vessel with 5 mL HNO_3_, 1 mL H_2_O_2_ and 2 mL HF. After 10 min, the samples were digested with a microwave digestion system (CEM, MARS-5, North Carolina, USA). The digestion procedure was as follows: the temperature gradually rose to 120 °C in 600 s and was maintained for 600 s, then rose to 160 °C in 480 s and was maintained for 600 s and finally rose to 180 °C in 480 s and was maintained for 600 s. The digestion residue was then transferred to an acid-cleaning Teflon tube and digested again at 90 °C using an electric heating panel for 4 h. Finally, the solution was diluted to 10 mL with deionized water, filtered through 0.45 μm filter and stored at −20 °C for testing.

The concentrations of Cr, Mn, Ni, Cu, Zn, As, Cd and Pb were determined by inductively coupled plasma mass spectrometry (ICP-MS, Agilent ICP-MS 7800, Santa Clara, USA) at the optimized condition [29].

### 2.4. Quality Assurance and Quality Control

To assure the quality of the pretreatment, the representative reference materials of blood (GBW(E)090033, Bovine blood, National Institute of Metrology, Beijing, China) and soil (GBW07407a, IGGE, Beijing, China) were included in each digestion batch. In addition, each digestion batch also contained 10% reagent blank sample and parallel sample to guarantee the quality. All chemicals were of guaranteed grade and the reagent blank was subtracted from the results.

The relative standard deviations in parallel measurements were lower than 5%. The recovery rates of elements ranged from 93% to 110%. The limits of detection (LOD) for all metals ranged from 2.0 to 20 μg·kg^−1^ (the details are presented in Table S1).

### 2.5. Exposure Assessment

Ingestion and dermal contact were the main exposure pathways for local children. Estimation of daily exposure dose (ng·kg^−1^ day^−1^) via hand wipe was based on the equations identified in previous studies [20,30], while the exposure dose via the outdoor soil and indoor dust referred to the models in the U.S. health risk guidelines [31].

The oral exposure doses via hand wipe, soil and dust ingestion were calculated using the Equations (1)–(3).

Ingestion via hand wipes:

(1) $${\mathrm{ADD}}_{oral}\;=\;\frac{Q_{hw}\;\times \;SA\;\times \;H_{contact\_area}\;\;\times \;TE\;\times \;f_{hm}\;\times \;t_{\exp}}{BW}$$

Ingestion via dust:

(2) $${\mathrm{ADD}}_{oral}\;=\;\frac{C_{dust}\;\times \;IR_{dust}\;\times \;f}{BW}$$

Ingestion via soil:

(3) $${\mathrm{ADD}}_{oral}\;=\;\frac{C_{soil}\;\times \;IR_{soil}\;\times \;f}{BW}$$

where *Q_hw_* is the metal mass adhering to hands per unit area (μg⸱m^−2^); *SA* is the hand skin surface area (m^2^); *H_contact-area_* is the proportion of hand surface area in each hand-to-mouth contact event (%); *TE* is the transfer efficiency of hand-to-mouth event (%); *f_hm_* is the hand-to-mouth frequency (times·h^−1^); *t_exp_* is the exposure time (h·day^−1^); *BW* is the body weight (kg); *C_dust_* is the metal concentration in dust (mg·kg^−1^); *IR_dust_* is the dust ingestion rate (mg·day^−1^); *f* is daily time proportion (%); *C_soil_* is the metal concentration in soil (mg·kg^−1^); *IR_soil_* is the soil ingestion rate (mg·day^−1^). Details of the parameter values are shown in Table S2 [32,33,34,35,36]. 

The hand-dermal exposure doses via hand wipe, soil and dust contact were calculated using Equations (4)–(6). 

Dermal absorption via hand wipes:

(4) $${\mathrm{ADD}}_{dermal}\;=\;\frac{Q_{hw}\;\;\times \;SA\;\times \;f_{hs}\;\times \;ABS}{BW}$$

Hand-dermal absorption via floor dust:

(5) $${\mathrm{ADD}}_{dermal}\;=\;\frac{C_{dust}\;\times \;DA\;\times \;SA\;\times \;f_{hs}\;\times \;ABS}{BW}$$

Hand-dermal absorption via soil:

(6) $${\mathrm{ADD}}_{dermal}\;=\;\frac{C_{soil}\;\times \;SD\;\times \;SA\;\times \;f_{hs}\;\times \;ABS}{BW}$$

where *DA* is the dust adherence factor (mg⸱cm^−2^); *f_hs_* is the daily exposure frequency (times⸱day^−1^*); SD* is the soil adherence factor (mg⸱cm^−2^); *ABS* is the dermal absorption factor (%). Details of the parameter values are shown in Table S2.

### 2.6. Risk Calculations

The hazard quotient (*HQ*) was used to characterize the non-carcinogenic risk, and the calculation equation recommended by the US. EPA was as follows [31].

(7) $$HQ\;=\;\frac{\mathrm{ADD}}{RfD}$$

where *RfD* is the maximum acceptable level at which an appreciable hazard to health is unlikely to occur over a lifetime in mg⸱kg^−1^ day^−1^). “*HQ* > 1” indicates that adverse health effect will happen. Details of the *RfD* for each target metal via oral and dermal contact pathway were presented in Table S3. Hazard index (*HI*) was calculated to characterize the accumulated non-carcinogenic risk associated with multiple metals and multiple routes.

(8) HI = ∑HQ

The carcinogenic risk was calculated using the following Equation (9) [37]:

(9) ILCR = ADD × SF

where *SF* is the cancer slope factor, which refers to the data from the Integrated Risk Information System (IRIS) of U.S. EPA. Details of the SF for each target metal via oral and dermal contact pathway are presented in Table S3. Risk lower than 10^−4^ was considered to be acceptable [38].

### 2.7. Statistic Analysis

The descriptive, difference, and correlation analyses were conducted using SPSS 20.0 software. A Kolmogorov–Smirnov test was adopted to evaluate the normality of the data. The difference analysis of metal concentration and exposure level among different sampling and evaluation campaigns was conducted by the Mann–Whitney U test. The relationship between external and internal exposure was conducted by the Spearman’s rank correlation analysis. Statistical significance was set at *p* < 0.05. In addition, sensitivity analysis was conducted by Monte Carlo simulation to analyze the contribution of each parameter to the total variance. 

## 3. Results and Discussions

### 3.1. Metal Concentrations in Different Sample Types

#### 3.1.1. Hand Wipes

The amounts of target metals in hand wipe samples per unit area are summarized in Table 1. It can be found that contaminant levels in hand wipes varied greatly among metals. Among the individual metals, Mn was the most predominant element (median = 280.2 μg⸱m^−2^), followed by Zn, Cr, Pb, Mn, Cu, Ni and As, with Zn, Cr and Pb one order of magnitude higher than Cu, Ni and As. Cd was the least abundant element in hand wipes, with a median value of 0.8 μg⸱m^−2^. Compared with the results from the limited published studies, the amount of Zn (199.5 μg⸱m^−2^), Cd (0.8 μg⸱m^−2^), As (21.5 μg⸱m^−2^) and Pb (101.4 μg⸱m^−2^) in the present study were much lower than the 4951 μg⸱m^−2^, 30.3 μg⸱m^−2^, 71.3 μg⸱m^−2^ and 2540 μg⸱m^−2^, respectively, for children after playing in the playground near the Port Pirie lead smelter from Broken Hill, Australia [39,40]. In addition, Pb levels (38,972 μg⸱m^−2^) for adults working at lead battery manufacturing sites in the UK were also much higher than that of the current study [41]. The difference could be largely explained by the relatively severe environment pollutant situation and the close human behavior (such as the occupational exposure) in those studies [42]. 

Intra-correlations between various metals in hand wipes were evaluated using Spearmen’s rank correlation coefficients (Table 2). Most metals showed positive and significant correlations with each other (*p* < 0.05). Some metals showed strong correlations (R ranges: 0.50–0.69), such as Ni and Cr (R = 0.69) and Pb and Mn (R = 0.61), whereas others were moderately correlated with each other (R ranges: 0.29–0.49), such as Cd and Cr (R = 0.29) and Cd and Mn (R = 0.39), indicating the similar pollution source. 

With the questionnaires, we further explored the potential environment and behavior factors which might be associated with metal levels in hand wipes (Table 3). It was found that the age of children (categorized into 3–6 years and 7–12 years; *n*= 30 each), years of residency (>5 years and ≤5 years; *n* = 14 and 46, respectively) and the ground types of children’s play areas (classified into bare soil and hard ground surface; *n* = 28 and 32, respectively) had effect on metal levels in hand wipes. In general, younger children (3–6 years) exhibited statistically higher metal levels on hands than did older children (7–12 years), except for Mn. This finding was consistent with the results from previous studies [43]. Significantly higher levels of most metals (including Cr, Mn, Zn, Cd and Pb) in hand wipes were found for children with years of residency higher than 5 years, in contrast with those who resided for a shorter time. Although the associations between residence time and metal levels on hands were not documented, residence times were identified to be the social factor significantly correlated with biological indicators such as blood Pb in previous studies [44]. Children, who played more frequently on bare soil had higher levels of Cr, Mn, Ni, Zn and Pb in hand wipes compared to those on hard ground surfaces. It suggested that the ground type determines the function of the floor as a sink for pollutants, which is especially true for metals with long environmental half-life [17]. Additionally, no statistical difference was found between hand-washing frequency and metal levels in hand wipes in this study. This finding was inconsistent with the results for organic pollutants [20,25,45], which reported that increased hand-washing frequency was associated with decreased amounts of PFOS and PRs in hand wipes. The possible explanation could be that metals had much lower octanol–air partition coefficients (*K_OA_*) in comparison with organic pollutants, while hand washing had a greater effect on hand-loading for volatile chemicals than for low-volatility chemicals [46].

#### 3.1.2. Comparison with Exterior Soil and Interior Dust

**Figure 1:**
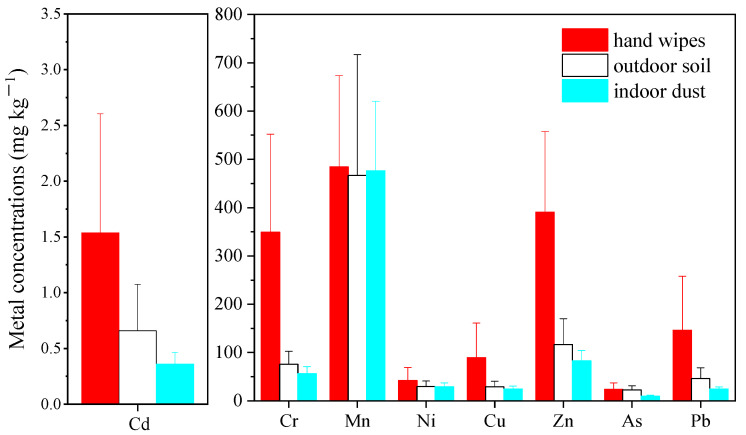
Comparison of metal concentrations via hand wipe, outdoor soil and indoor dust.

To compare the metal levels in hand wipes with those in other matrixes, metal concentrations in hand wipes were obtained through dividing the amounts of metals on hands by the mass of hand dust loading. This is the first study exhibiting the metal concentrations in hand wipes in mg⸱kg^−1^. The comparison of metal concentrations in various sampling matrixes are shown in Figure 1.

It was observed that metal concentrations differed greatly among paired hand wipes and outdoor soil or indoor dust. For most metals, including Cr, Ni, Cu, Zn, Cd and Pb, hand wipes had significantly higher concentrations than those of outdoor soil and indoor dust (*p* < 0.05). For instance, Cr levels in hand wipes were five times of those in dust and six times of those in soil, whereas no significant difference was observed for Mn among these three matrixes. The As levels in hand wipes were slightly higher than in dust but threefold higher than in soil. In addition, different profiles of metal concentrations were also observed across these three matrixes, especially for Mn, Zn and Cr (Figure S1). The contribution rates of Mn to the total metal concentrations in soil and dust were approximately two times those in hand wipes, while Zn and Cr contributed two to three times higher proportions in hand wipes than in soil and dust.

One possible explanation for the distinctions among the different matrixes for diverse metals could be that, in addition to the sampling site, participants might go to other microenvironments, such as vehicles, school and so on. For instance, nationwide surveys found that children aged 3 to 12 years generally spent 23 to 42 min in vehicles per day [47,48], and a third to a quarter of indoor activity time was spent somewhere other than home [49]. Thus, the hand-adhered soil could be derived from the soil or dust in other microenvironments, which may have different metal levels and profiles in comparison with the sampled site due to the different pollution sources.

Furthermore, the difference could be associated with the discrepancy of particle size of the three matrixes and the heterogeneity of metal distribution in different soil particle fractions. In one aspect, it has been confirmed that finer soil/dust particles tended to adhere more efficiently to human hands. For instance, Ikegami et al. [50] found that approximately 90% of soil particles on hands were smaller than 100 μm. In addition, the particle size of hand soil was susceptible to the factors of soil texture (such as soil type and soil moisture content) and human behavior (such as the way hands contact soil or dust) [27,51]. Thus, the particle size of the soil adhering to hands could be larger than that of the outdoor soil and indoor dust and may vary among individuals. In the other aspect, metals were not homogeneously distributed among soil particle fractions. Most metals were inclined to accumulate in higher concentrations in finer fractions [52,53]. Therefore, metals in hand wipes exhibited higher concentrations than those in outdoor soil and indoor dust. In comparison with the single-soil or dust sampling, the hand wipe was a more direct sampling method which could better represent the entire exposure from multiple microenvironments and the real particle size to which humans are exposed.

### 3.2. Exposure and Risk Level

#### 3.2.1. Hand Wipes

Human exposure to metals on hands could occur through hand-to-mouth contact and hand-dermal adsorption. Children’s exposures via each pathway were assessed based on measured metal mass in hand wipes and personalized exposure parameters from the questionnaires (Table 4). Due to the low dermal absorption factor of metals, the exposure dose through dermal absorption was approximately two to three orders of magnitude lower than that via ingestion. Thus, for all metals, ingestion was the dominant exposure pathway, accounting for nearly 99% of the total exposure.

The median combined HI of the target metals was within the acceptable level (Table S4), whereas the HI was 1.5 for the high-end scenario (95th percentile), indicating the potential non-carcinogenic risk to a small portion of the local children. The hand-to-mouth pathway was the dominant risk source, accounting for 74% to 99% of the total risk. The risk decreased in the order of Cr > As > Pb > Mn > Ni > Cu > Zn > Cd. Although Cr was not the most abundant toxic elements on hands, it contributed most to the total non-carcinogenic risk because of its high toxicity.

The total cancer risk was 4.0 × 10^−4^ (Table S5), indicating the potential health threat to the local children. Cr contributed 63% to the accumulative risk, with its individual risk already exceeding the maximum acceptable level. Oral ingestion through hand-to-mouth contact was the dominant exposure pathway, accounting for 89% of the total risk.

In addition, sensitive analysis was conducted using the Monte Carlo simulation with Cr oral exposure via hand-to-mouth contact as an example to quantitatively evaluate the contribution of each parameter to the total variance of risk level (Figure S2). It can be observed that metal mass on hand wipes was the dominant factor, which contributed nearly half of the total variance, followed by body weight. In addition, the contribution of 23.1% deriving from hand-to-mouth contact frequency should not be neglected, implying the importance of using personalized data during exposure assessment.

#### 3.2.2. Comparison with Exterior Soil and Interior Dust

**Figure 2:**
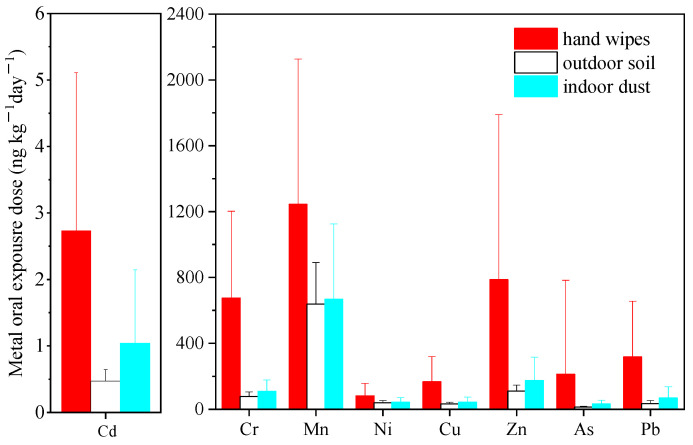
Comparison of metal oral exposure dose via hand wipes, outdoor soil and indoor dust.

**Figure 3:**
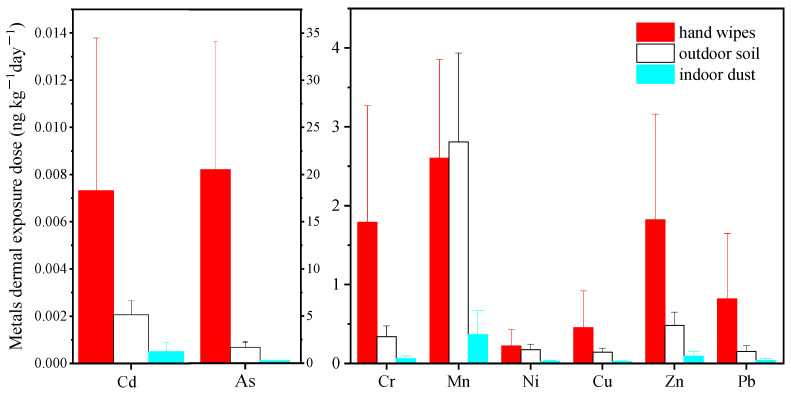
Comparison of metal hand-dermal exposure dose via hand wipe, outdoor soil and indoor dust.

The comparisons of children’s metal exposure via oral ingestion and hand-dermal adsorption pathways using different sampling and assessing strategies are displayed in Figure 2 and Figure 3, respectively. It was observed that when using the data from different sampling campaigns and corresponding assessment models, the estimated exposure dose differed greatly for most metals.

In terms of ingestion pathway, the exposures to most metals (including Cr, Cu, Zn, As, Cd and Pb) via hand wipes were 5–17 times greater than the estimation via soil and 4–7 times higher than that via dust. However, for Mn and Ni, the oral exposure dose using the hand wipe method provided good agreement with those using the dust or soil sampling approach (*p* > 0.05). While for the dermal absorption pathway, even greater discrepancies were observed. For Cr, Cu, Zn, As, Cd and Pb, the estimated dermal exposure dose via hand wipes were 3–12 times greater than those via soil (*p* < 0.05) and 14–40 times greater than those via dust (*p* < 0.05), whereas for Mn and Ni, there were no significant differences between the dermal exposure dose through hand wipes and soil, while 7 to10 times higher exposure was observed for hand wipes compared to dust.

In addition, the discrepancy in health risk among these three methods was similar to that in exposure dose (Tables S6–S9). Remarkably, the cancer risk deriving from Cr exposure via hand wipes had already exceeded the acceptable threshold, while it was acceptable when using the soil and dust sampling methods.

The inconsistency in exposure and risk levels among the three sampling strategies was associated with not only the difference in exposure concentrations but also the discrepancy in assessment models. Different exposure parameters were involved in different models, such as the hand-to-mouth contact frequency in the hand wipes model, the soil ingestion rate and soil adherence factor in the soil model, and the dust ingestion rate and dust adherence factor in the dust model. It is worth noting that fixed values deriving from studies about limited participants were assigned for the parameters for exposure assessment using data from soil and dust sampling campaigns, including the soil/dust ingestion rate in the ingestion model, and soil/dust adherence factor in the dermal absorption model. These parameters were hard to individualize and could be influenced by factors such as soil or dust properties and personal behaviors [53,54]. Significant discrepancies were already observed between the assessment of organophosphate flame retardant exposure using personalized data and that used the fixed values from the general population [55]. Therefore, the usage of fixed values was insufficient to reflect the real exposure and the individual variance. In contrast, personalized data for parameters, including hand-to-mouth contact and hand soil loading, were introduced in the hand wipe model, and thus the true extent of the exposure and the difference across participants could be well reflected.

### 3.3. Association between External and Internal Exposure

To further compare the suitability of the three sampling strategies, the associations between metal levels in the external environment and blood were explored (Table S10). Metal levels in blood were comparable to those reported among the general Chinese population [56]. The average Pb level was 3.2 μg·dL^−1^, with 89 % children’s levels lower than the 5 μg·dL^−1^ recommended by the US Centers for Disease Control and Prevention.

**Figure 4:**
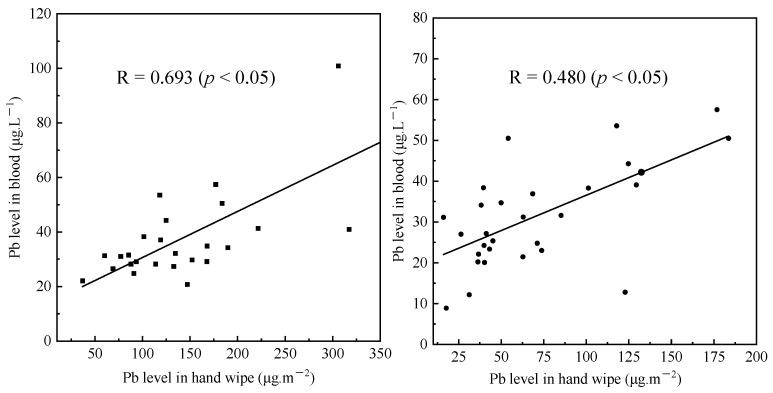
Correlations of Pb level in hand wipe and blood (left: children aged 3–6 years; right: children aged 7–12 years).

A strong correlation was found between hand wipe Pb and blood Pb (R = 0.534, *p* < 0.05), whereas Pb in soil and dust showed no significant correlations with that in blood, indicating the hand wipe pathway is an important contributor to Pb exposure. It also suggested that hand wipe Pb could be a better predictor of blood Pb in comparison with soil and dust. Interestingly, the extent of the correlation decreased with age. For example, the Spearmen’s rank correlation coefficient for hand wipe Pb and blood Pb was 0.693 for children aged 3–6 years (*p* < 0.05), while the correlation coefficient reduced to 0.480 for children aged 7–12 years (*p* < 0.05) (Figure 4). A possible explanation could be that children’s hand-to-mouth contact frequency decreased with age [46,47], and thus the corresponding contribution to the total exposure might be reduced with advancing age. The significant relationship between Pb in blood and hand wipes was also found for children younger than 3 years by Gulson et al. [57]. In addition, Gulson et al. [58] found that the predicted blood Pb matched better with the observed values for children younger than 6 years when using hand wipe Pb instead of dust Pb in the IEUBK model. These all demonstrated the suitability of hand wipe Pb as a predictor of internal exposure level.

In addition, Cr and Mn in blood correlated significantly and moderately with those in hand wipes. Likewise, no associations with exterior soil and interior dust were found. It suggested that hand-to-mouth contact was a non-negligible pathway for Cr and Mn exposure. A similar relationship was also observed by Gulson et al. [56] for Mn. Actually, in previous multiple exposure pathways studies, diet was found to be the largest contributor to Mn exposure, while soil only contributed 5% to 30% to the total exposure [13,14], whereas another study found that Mn exhibited stronger correlations with hand wipes than with diet [57]. The possible explanation could be: (1) soil sampling rather than hand wipe sampling strategies were used in those studies, which might underestimate the soil exposure to some extent and (2) the bioavailability was always overlooked in health risk assessment, while it varied among food and soil [51,56]. No correlations were found for other metals, which might be due to other exposure pathways (such as food, water and air) having more obvious contributions to the total exposure.

### 3.4. Implication for Sampling and Assessing Method Selection

Human exposure to metals differed greatly based on hand wipe, indoor dust and outdoor soil, indicating the importance of method selection when assessing human metal exposure. Our study suggests that hand wipes could serve as a better matrix of soil/dust exposure compared to the single-soil or dust sample. On one hand, hand wipes could reflect the combined exposure to all microenvironments visited by the subject during the whole sampling period, while soil or dust sampled from a single location is only influenced by the adjacent pollution source. On the other hand, hand wipes could reflect the real particle size of soil or dust to which the subject is exposed, while soil/dust-sieving strategies varied greatly among studies, leading to the bias in assessment results and the incomparability across studies. The superiority of using hand wipes as the matrix for the evaluation of human exposure to metals could also be supported by the taking into account the personalized data in the corresponding exposure assessment model, thus individual difference could be better reflected. In addition, hand wipes exhibited significant association with internal exposure.

However, it is worth noting that information on hand wipes might be affected by several factors, such as the sampling time. A previous study found that Mn in hand wipes in autumn were significantly lower than in winter [58]. Thus, the standardization of the sampling methods needs to be studied. Hand-washing frequency was also identified to be another influencings factor for hand organic pollutants [20,25,44], while no relationship was observed for metals in the present study. Moreover, hand wipes only evaluated the exposure through the hand-to-mouth contact and hand-dermal adsorption pathways, whereas other potential pathways, such as food ingestion, were not included. Even so, hand wipes still yielded good correlations with blood, indicating their important contribution to metals exposure. Since hand wipes could only assess the dermal exposure via hand contact, the representativeness of exposure from other routes thus needs to be further studied.

### 3.5. Strengths and Limitations

Major strengths of the study included that a comprehensive comparison of the sampling strategy combing the discrepancy in external exposure and the association with internal exposure was employed. In addition, personalized data was analyzed during the assessment to reflect the individual variance.

There were several limitations to the study. First, a relatively small sample size was involved in this study. Second, metal bioavailability was not considered in the study, which may lead to the overestimation of the exposure risk to some extent. Third, the object of the study was children younger than 12 years, thus the applicability for other population needs to be further studied. In addition, due to the unavailability of the toxic efficiency for bulk Cr, the RfD for Cr (VI) was employed in the current study as a surrogate, which may overestimate the corresponding exposure risk to a certain extent [59].

## 4. Conclusions

To the best of our knowledge, this study provided the first chance to comprehensively compare the difference in metal exposure based on hand wipes, indoor dust and outdoor soil, combining the methods of external exposure assessment and the correlation with internal exposure level. The oral exposure cancer risk to Cr through hand-to-mouth contact even exceeded the maximum acceptable level, emphasizing the importance of metal exposure via the hand wipe pathway. Using different sampling and assessing strategies could lead to differences of several to dozens of times in human exposure outcomes. In addition, Pb, Mn and Cr in hand wipes were significantly correlated with those in blood, whereas no relationships were found with soil and dust. In comparison with soil and dust, hand wipes served as a better matrix to assess human metal exposure because it could reflect the true and integrated exposure levels and the individual differences. Further studies should standardize the sampling method of hand wipes and verify its applicability for other populations.

## Figures and Tables

**Table 1:** Descriptive statistics of the amounts of metals in hand wipe samples per unit area.

Metals	Hand Wipes (μg·m^−2^)				
	Mean	Std	Median	P5	P95
Cr	242.2	173.9	184.1	96.6	693.4
Mn	373.4	275.0	280.2	105.1	912.9
Ni	29.8	23.8	21.4	9.9	90.5
Cu	61.2	52.3	43.2	18.0	169.5
Zn	258.4	202.4	199.5	84.7	600.7
As	23.6	13.5	21.5	6.2	48.4
Cd	1.0	0.7	0.8	0.3	2.4
Pb	106.9	87.7	101.4	26.4	305.7

**Table 2:** Spearmen’s rank correlations between metals in hand wipes.

Metals	Cr	Mn	Ni	Cu	Zn	As	Cd	Pb
Cr	1							
Mn	0.39 **	1						
Ni	0.69 **	0.49 **	1					
Cu	0.47 **	0.26	0.68 **	1				
Zn	0.54 **	0.57 **	0.48 **	0.41 **	1			
As	0.59 **	0.59 **	0.48 **	0.44 **	0.53 **	1		
Cd	0.29 *	0.39 **	0.53 **	0.42 **	0.21	0.30 *	1	
Pb	0.12	0.61 **	0.28	0.29 *	0.37 **	0.41 **	0.17	1

** Correlation is significant at the 0.01 level (2-tailed). * Correlation is significant at the 0.05 level (2-tailed).

**Table 3:** Analysis of the influencing factors on metal levels in hand wipes (μg·m^−2^).

Influencing Factors	*n*	Cr	Mn	Ni	Cu	Zn	As	Cd	Pb
Age of children									
3–6 years	30	294.4	443.8	31.8	79.4	389.5	32.9	1.1	151.9
7–12 years	30	164.5	184.4	18.7	39.6	177.3	11.1	0.7	59.9
*p*		<0.05	0.11	<0.05	<0.05	<0.05	<0.05	<0.05	<0.05
Years of residency									
>5 years	14	226.3	397.9	21.4	44.7	253.4	18.5	0.9	107.4
≤5 years	46	171.0	180.9	18.5	41.7	146.7	11.6	0.6	62.0
*p*		<0.05	<0.05	0.27	0.85	<0.05	0.36	<0.05	<0.05
Ground types of children’s regular play areas									
Bare soil	28	266.3	443.8	31.4	44.7	244.4	15.5	1.2	107.4
Hard ground surface	32	150.1	184.4	11.3	41.6	123.7	14.2	0.8	65.7
*p*		<0.05	<0.05	<0.05	0.61	<0.05	0.58	0.80	<0.05
Hand-washing frequency									
>5 times⸱d^−1^	33	180.4	241.4	21.4	49.0	200.8	11.9	0.9	72.5
≤5 times⸱d^−1^	27	226.3	376.8	21.3	41.5	193.6	16.2	0.7	88.0
*p*		0.66	0.26	0.79	0.55	0.40	0.82	0.71	0.98

**Table 4:** The estimated exposure dose to metals in hand wipes through hand-to-mouth contact and dermal absorption pathways.

Metals	Hand-to-Mouth Contact (ng kg^−1^ day^−1^)					Dermal Absorption (ng kg^−1^ day^−1^)				
	Mean	Std	Median	P5	P95	Mean	Std	Median	P5	P95
Cr	674.9	627.7	452.1	117.0	1662.1	1.8	1.5	1.3	0.4	5.3
Mn	1244.2	883.0	982.7	135.2	2485.5	2.6	2.3	2.2	0.5	7.5
Ni	80.9	76.7	56.6	14.0	192.0	0.2	0.2	0.1	0.0	0.7
Cu	166.8	152.9	101.9	24.6	504.8	0.5	0.5	0.3	0.1	1.2
Zn	787.2	502.0	445.2	131.0	1299.0	1.8	1.3	1.4	0.3	4.9
As	212.2	170.7	22.9	2.1	825.0	20.5	13.5	3.3	1.0	86.5
Cd	2.7	2.4	1.9	0.5	6.1	0.007	0.006	0.005	0.002	0.018
Pb	318.3	237.4	149.3	40.7	1070.5	0.8	0.8	0.6	0.1	2.7

## Supplementary Materials

The following supporting information can be downloaded at: https://www.mdpi.com/article/10.3390/ijerph192114614/s1, Table S1 Instrument and method detection limits for different metals in the study; Table S2 The value of parameters in exposure assessment via different sampling and evaluation strategies; Table S3 Summary of toxicity factors used for risk assessment; Table S4 The non-carcinogenic risk form exposure to metals in hand wipes through hand to mouth contact and dermal absorption pathway; Table S5 The cancer risk form exposure to metals in hand wipes through hand to mouth contact and dermal absorption pathway; Table S6 The non-carcinogenic risk form exposure to metals in outdoor soil through ingestion and dermal absorption pathway; Table S7 The cancer risk form exposure to metals in outdoor soil through ingestion and dermal absorption pathway; Table S8 The non-carcinogenic risk form exposure to metals in indoor dust through ingestion and dermal ab-sorption pathway; Table S9 The cancer risk form exposure to metals in indoor dust through in-gestion and dermal absorption pathway; Table S10 Spearmen’s Rank Correlations coefficient of metals concentrations in blood and in external exposure concentration; Figure S1. Comparison of metals concentration profile in hand wipes, indoor dust and outdoor soil; Figure S2 the Sensitivity analysis of Cr oral exposure from hand wipe via hand to mouth pathway.
