# Supplementary material for: Assessment of Children’s Metal Exposure via Hand Wipe, Outdoor Soil and Indoor Dust and Their Associations with Blood Biomarkers
Source: Int J Environ Res Public Health. 2022 Nov 7;19(21):14614. doi: 10.3390/ijerph192114614 (PMC9653965; doi:10.3390/ijerph192114614)
Supplement: Supplementary file 1 [file ijerph-19-14614-s001.zip › ijerph-1977348-supplementary.pdf]

# Supporting Information

**Assessment of children's metals exposure via hand wipe, outdoor soil and indoor dust, and their associations with blood biomarkers: comparison of different evaluation campaigns**

Wang Beibei [a], Gao Fei [a], Lin Chunye [b], Cheng Hongguang [b], Ma Jin [c], Duan Xiaoli [a,*]

[a] School of Energy and Environmental Engineering, University of Science and Technology Beijing, Beijing, 100083, PR China

[b] State Key Joint Laboratory of Environmental Simulation and Pollution Control, School of Environment, Beijing Normal University, Beijing, 100875, PR China

[c] State Key Laboratory of Environmental Criteria and Risk Assessment, Chinese Research Academy of Environmental Sciences, Beijing, 100012, PR China

**Table S1**. Instrument and method detection limits for different metals in the study

| Metals | Detection limit (μg·kg$^{-1}$) | RSD% | Recovery% |
|---|---|---|---|
| Pb | 8.0 | 2.65 | 94.81 |
| Cr | 10.0 | 3.01 | 96.43 |
| Cd | 5.0 | 2.18 | 95.34 |
| Mn | 15.0 | 2.31 | 97.32 |
| Ni | 10.0 | 1.56 | 104.51 |
| Cu | 10.0 | 4.11 | 106.31 |
| Zn | 20.0 | 2.81 | 102.52 |
| As | 4.0 | 3.62 | 101.36 |

**Table S2.** The value of parameters in exposure assessment via different sampling and evaluation strategies.

| arameters | values | reference |
|---|---|---|
| $Q_{hw}$, $C_{dust}$, $C_{soil}$ | personalized | According to the sampling and measurement |
| $H_{contact\text{-}area}$ | 0.1 | Stapleton et al. (2008) |
| $TE$ | 0.5 | kissel et al. (1998) |
| $f_{hm}$ | personalized | According to questionnaire results |
| $t_{exp}$ | 12 | Ma et al. (2018) |
| $BW$ | personalized | According to questionnaire results |
| $IR_{dust}$ | 72 mg/day (3-6) | Lin et al. (2017) |
| | 103 mg/day (7-12) | Wang et al. (2018) |
| $IR_{soil}$ | 72 mg/day (3-6) | Lin et al. (2017) |
| | 103 mg/day (7-12) | Wang et al. (2018) |
| $f$ | 0.5 | Liu et al. (2018) |
| $SD$ | 0.099 mg/cm$^2$ | Kissel et al. (1996) |
| $DA$ | 0.011 mg/cm$^2$ | Holmes et al. (1999) |
| $f_{hs}$ | personalized | According to questionnaire results |
| $ABS$ | As: 0.03 | |
| | Others：0.001 | U.S.EPA 2004 |
| $SA$ | personalized | According to questionnaire results |

**Table S3**. Summary of toxicity factors used for risk assessment

| Contaminant of potential concern | Oral RfD ($mg \cdot kg^{-1} day^{-1}$) | Dermal RfD ($mg \cdot kg^{-1} day^{-1}$) | Oral SF ($(mg \cdot kg^{-1} day^{-1})^{-1}$) | Dermal SF ($(mg \cdot kg^{-1} day^{-1})^{-1}$) |
|---|---|---|---|---|
| Pb | 3.50E-03 | 5.25E-04 | NA | NA |
| Cr | 3.00E-03* | 3.00E-03* | 5.01E-01 | 2.00E+01 |
| Cd | 1.00E-03 | 1.00E-05 | NA | NA |
| Mn | 1.40E-01 | 2.33E-02 | NA | NA |
| Ni | 2.00E-02 | 5.40E-03 | 1.70E+00 | 4.25E+01 |
| Cu | 4.00E-02 | 1.20E-02 | NA | NA |
| Zn | 3.00E-01 | 6.00E-02 | NA | NA |
| As | 3.00E-04 | 1.23E-04 | 1.50E+00 | 3.66E+00 |

NA—not applicable

*—the RfD is for Cr (VI)

**Table S4**. The non-carcinogenic risk form exposure to metals in hand wipes through hand to mouth contact and dermal absorption pathway.

| Metals | hand to mouth contact | | | Dermal absorption | | | Total | | |
|---|---|---|---|---|---|---|---|---|---|
| | Median | P5 | P95 | Median | P5 | P95 | Median | P5 | P95 |
| Cr | 1.5E-01 | 3.9E-02 | 5.5E-01 | 4.4E-04 | 1.5E-04 | 1.8E-03 | 1.5E-01 | 3.9E-02 | 5.6E-01 |
| Mn | 4.9E-03 | 9.7E-04 | 3.2E-02 | 9.6E-05 | 2.0E-05 | 3.2E-04 | 5.0E-03 | 9.9E-04 | 3.2E-02 |
| Ni | 2.8E-03 | 7.0E-04 | 9.6E-03 | 2.6E-05 | 8.8E-06 | 1.3E-04 | 2.9E-03 | 7.1E-04 | 9.7E-03 |
| Cu | 2.5E-03 | 6.1E-04 | 1.3E-02 | 2.2E-05 | 9.2E-06 | 1.0E-04 | 2.6E-03 | 6.2E-04 | 1.3E-02 |
| Zn | 1.5E-03 | 4.4E-04 | 7.7E-03 | 2.3E-05 | 5.6E-06 | 8.2E-05 | 1.5E-03 | 4.4E-04 | 7.7E-03 |
| As | 7.6E-02 | 7.1E-03 | 4.4E-01 | 2.7E-02 | 8.0E-03 | 1.4E-01 | 1.0E-01 | 1.5E-02 | 5.8E-01 |
| Cd | 1.9E-03 | 4.7E-04 | 6.1E-03 | 5.2E-04 | 1.6E-04 | 1.8E-03 | 2.4E-03 | 6.3E-04 | 7.9E-03 |
| Pb | 4.3E-02 | 1.2E-02 | 3.1E-01 | 1.1E-03 | 2.6E-04 | 5.1E-03 | 4.4E-02 | 1.2E-02 | 3.1E-01 |
| Sum | 2.8E-01 | 6.1E-02 | 1.4E+00 | 2.9E-02 | 8.6E-03 | 1.4E-01 | 3.1E-01 | 7.0E-02 | 1.5E+00 |

**Table S5.** The cancer risk form exposure to metals in hand wipes through hand to mouth contact and dermal absorption pathway.

| Metals | Hand to mouth contact | | | Dermal absorption | | | Total | | |
|---|---|---|---|---|---|---|---|---|---|
| | Median | P5 | P95 | Median | P5 | P95 | Median | P5 | P95 |
| Cr | 2.3E-04 | 5.9E-05 | 8.3E-04 | 2.6E-05 | 8.8E-06 | 1.1E-04 | 2.5E-04 | 6.7E-05 | 9.4E-04 |
| Ni | 9.6E-05 | 2.4E-05 | 3.3E-04 | 5.9E-06 | 2.0E-06 | 3.1E-05 | 1.0E-04 | 2.6E-05 | 3.6E-04 |
| As | 3.4E-05 | 3.2E-06 | 2.0E-03 | 1.2E-05 | 3.6E-06 | 6.1E-04 | 4.6E-05 | 6.8E-06 | 2.6E-03 |
| Sum | 3.6E-04 | 8.6E-05 | 3.1E-03 | 4.4E-05 | 1.4E-05 | 7.5E-04 | 4.0E-04 | 1.0E-04 | 3.9E-03 |

**Table S6**. The non-carcinogenic risk form exposure to metals in outdoor soil through ingestion and dermal absorption pathway.

| Metals | Soil ingestion | | | Dermal absorption | | | Total | | |
|---|---|---|---|---|---|---|---|---|---|
| | Median | P5 | P95 | Median | P5 | P95 | Median | P5 | P95 |
| Cr | 2.4E-02 | 8.7E-03 | 4.2E-02 | 1.0E-04 | 4.5E-05 | 1.9E-04 | 2.4E-02 | 8.7E-03 | 4.2E-02 |
| Mn | 4.5E-03 | 1.6E-03 | 7.4E-03 | 1.3E-04 | 5.0E-05 | 2.0E-04 | 4.6E-03 | 1.6E-03 | 7.6E-03 |
| Ni | 2.1E-03 | 7.1E-04 | 2.9E-03 | 3.2E-05 | 1.4E-05 | 5.5E-05 | 2.1E-03 | 7.3E-04 | 2.9E-03 |
| Cu | 8.5E-04 | 3.3E-04 | 1.2E-03 | 1.1E-05 | 6.3E-06 | 2.0E-05 | 8.7E-04 | 3.4E-04 | 1.2E-03 |
| Zn | 3.8E-04 | 1.5E-04 | 5.3E-04 | 7.6E-06 | 4.7E-06 | 1.4E-05 | 3.9E-04 | 1.5E-04 | 5.4E-04 |
| As | 4.1E-02 | 1.6E-02 | 7.5E-02 | 1.3E-02 | 5.9E-03 | 2.2E-02 | 5.4E-02 | 2.2E-02 | 9.7E-02 |
| Cd | 4.7E-04 | 2.1E-04 | 7.5E-04 | 2.0E-04 | 9.3E-05 | 3.2E-04 | 6.7E-04 | 3.1E-04 | 1.1E-03 |
| Pb | 8.0E-03 | 3.2E-03 | 1.9E-02 | 2.3E-04 | 1.1E-04 | 5.6E-04 | 8.2E-03 | 3.3E-03 | 2.0E-02 |
| Sum | 8.2E-02 | 3.1E-02 | 1.5E-01 | 1.4E-02 | 6.3E-03 | 2.3E-02 | 9.5E-02 | 3.7E-02 | 1.7E-01 |

Table S7 The cancer risk form exposure to metals in outdoor soil through ingestion and dermal absorption pathway.

| Metals | Soil ingestion | | | Dermal absorption | | | Total | | |
|---|---|---|---|---|---|---|---|---|---|
| | Median | P5 | P95 | Median | P5 | P95 | Median | P5 | P95 |
| Cr | 3.7E-05 | 1.3E-05 | 6.3E-05 | 6.1E-06 | 2.7E-06 | 1.2E-05 | 4.3E-05 | 1.6E-05 | 7.5E-05 |
| Ni | 7.0E-05 | 2.4E-05 | 9.8E-05 | 7.3E-06 | 3.1E-06 | 1.3E-05 | 7.7E-05 | 2.7E-05 | 1.1E-04 |
| As | 1.8E-05 | 7.1E-06 | 3.4E-05 | 5.8E-06 | 2.7E-06 | 1.0E-05 | 2.4E-05 | 9.8E-06 | 4.4E-05 |
| Sum | 1.3E-04 | 4.4E-05 | 1.9E-04 | 1.9E-05 | 8.5E-06 | 3.4E-05 | 1.4E-04 | 5.3E-05 | 2.3E-04 |

**Table S8**. The non-carcinogenic risk form exposure to metals in indoor dust through ingestion and dermal absorption pathway.

| Metals | Dust ingestion | | | Dermal absorption | | | Total | | |
|---|---|---|---|---|---|---|---|---|---|
| | Median | P5 | P95 | Median | P5 | P95 | Median | P5 | P95 |
| Cr | 3.4E-02 | 7.6E-03 | 7.2E-02 | 1.7E-05 | 5.3E-06 | 3.2E-05 | 3.4E-02 | 7.6E-03 | 7.2E-02 |
| Mn | 3.3E-03 | 4.7E-04 | 1.3E-02 | 1.2E-05 | 2.6E-06 | 3.7E-05 | 3.3E-03 | 4.7E-04 | 1.3E-02 |
| Ni | 2.0E-03 | 4.5E-04 | 4.2E-03 | 3.7E-06 | 1.2E-06 | 6.8E-06 | 2.0E-03 | 4.5E-04 | 4.2E-03 |
| Cu | 8.5E-04 | 1.9E-04 | 2.3E-03 | 1.7E-06 | 4.7E-07 | 3.6E-06 | 8.5E-04 | 1.9E-04 | 2.3E-03 |
| Zn | 5.3E-04 | 8.6E-05 | 1.2E-03 | 1.4E-06 | 3.3E-07 | 4.0E-06 | 5.3E-04 | 8.6E-05 | 1.2E-03 |
| As | 1.1E-01 | 2.7E-02 | 2.4E-01 | 3.9E-03 | 1.1E-03 | 7.8E-03 | 1.2E-01 | 2.8E-02 | 2.5E-01 |
| Cd | 8.5E-04 | 1.2E-04 | 2.3E-03 | 4.5E-05 | 1.0E-05 | 1.6E-04 | 9.0E-04 | 1.3E-04 | 2.4E-03 |
| Pb | 1.2E-02 | 5.3E-03 | 6.2E-02 | 6.4E-05 | 2.1E-05 | 1.1E-04 | 1.2E-02 | 5.3E-03 | 6.2E-02 |
| Sum | 1.7E-01 | 4.1E-02 | 3.9E-01 | 4.1E-03 | 1.2E-03 | 8.2E-03 | 1.7E-01 | 4.3E-02 | 4.0E-01 |

**Table S9.** The cancer risk form exposure to metals in indoor dust through ingestion and dermal absorption pathway.

| Metals | Dust ingestion | | | Dermal absorption | | | Total | | |
|---|---|---|---|---|---|---|---|---|---|
| | Median | P5 | P95 | Median | P5 | P95 | Median | P5 | P95 |
| Cr | 5.1E-05 | 1.1E-05 | 1.1E-04 | 1.0E-06 | 3.2E-07 | 1.9E-06 | 5.2E-05 | 1.2E-05 | 1.1E-04 |
| Ni | 6.7E-05 | 1.5E-05 | 1.4E-04 | 8.4E-07 | 2.7E-07 | 1.6E-06 | 6.8E-05 | 1.6E-05 | 1.4E-04 |
| As | 5.1E-05 | 1.2E-05 | 1.1E-04 | 1.8E-06 | 5.1E-07 | 3.5E-06 | 5.3E-05 | 1.3E-05 | 1.1E-04 |
| Sum | 1.7E-04 | 3.9E-05 | 3.6E-04 | 3.6E-06 | 1.1E-06 | 7.0E-06 | 1.7E-04 | 4.0E-05 | 3.6E-04 |

**Table S10.** Spearmen's Rank Correlations coefficient of metals concentrations in blood and in external exposure concentration.

| Metals | blood VS hand wipe | blood VS outdoor soil | blood VS indoor dust |
|---|---|---|---|
| Cr | **0.416*** | 0.264 | 0.214 |
| Mn | **0.339*** | 0.327 | 0.055 |
| Ni | 0.05 | 0.391 | 0.267 |
| Cu | 0.195 | 0.445 | 0.233 |
| Zn | 0.016 | 0.109 | 0.393 |
| As | 0.064 | 0.375 | 0.427 |
| Cd | 0.023 | 0.173 | 0.035 |
| Pb | **0.534**** | 0.152 | 0.154 |

**Correlation is significant at the 0.01 level (2-tailed)

* Correlation is significant at the 0.05 level (2-tailed)

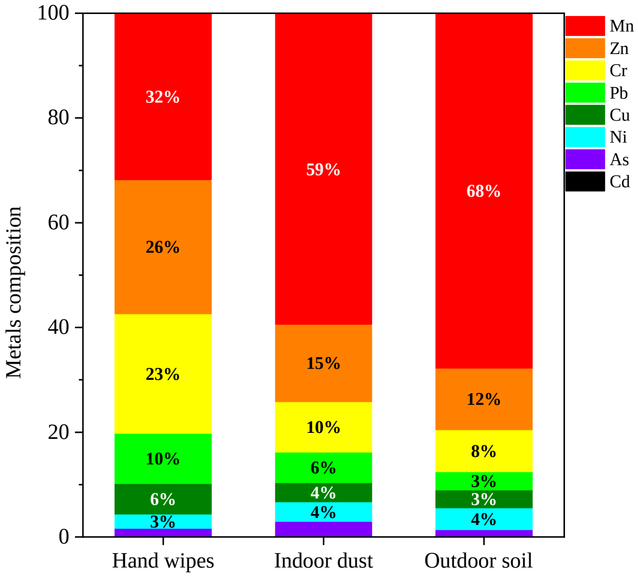


**Figure S1**. Comparison of metals concentration profile in hand wipes, indoor dust and outdoor soil

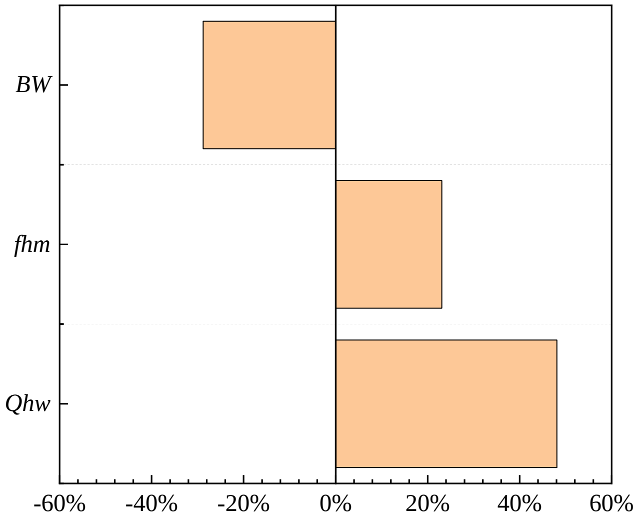


**Figure S2** the Sensitivity analysis of Cr oral exposure from hand wipe via hand to mouth pathway.

References:

Holmes, K.K., Shirai, J.H., Richter, K.Y., Kissel, J.C., 1999. Field measurement of dermal soil loadings in occupational and recreational activities. Environmental Research 80,(2), 148-157. https://doi.org/10.1006/enrs.1998.3891

Kissel, J.C., Richter, K.Y., Fenske, R.A., 1996. Factors affecting soil adherence to skin in hand-press trials. Bulletin of Environmental Contamination and Toxicology 56,(5), 722-728. https://doi.org/10.1007/s001289900106

Kissel, J.C., Shirai, J.H., Richter, K.Y., Fenske, R.A., 1998. Empirical investigation of hand-to-mouth transfer of soil. Bulletin of Environmental Contamination and Toxicology 60,(3), 379-386. https://doi.org/10.1007/s001289900637

Lin, C., Wang, B., Cui, X., Xu, D., Cheng, H., Wang, Q., Ma, J., Chai, T., Duan, X., Liu, X., Ma, J., Zhang, X., Liu, Y., 2017. Estimates of Soil Ingestion in a Population of Chinese Children. Environ Health Perspect 125,(7), 077002. https://doi.org/10.1289/EHP930

Ma, J., Pan, L.B., Wang, Q., Lin, C.Y., Duan, X.L., Hou, H., 2018. Estimation of the daily soil/dust (SD) ingestion rate of children from Gansu Province, China via hand-to-mouth contact using tracer elements. Environmental Geochemistry and Health 40,(1), 295-301. https://doi.org/10.1007/s10653-016-9906-1

Stapleton, H.M., Kelly, S.M., Allen, J.G., McClean, M.D., Webster, T.F., 2008. Measurement of

polyhrominated diphenyl ethers on hand wipes: Estimating exposure from hand-to-mouth contact. Environmental Science & Technology 42,(9), 3329-3334. https://doi.org/10.1021/es7029625

U.S.EPA, 2004. Risk Assessment Guidance for Superfund Volume I: Human Health Evaluation Manual (Part E, Supplemental Guidance for Dermal Risk Assessment). U.S. Environmental Protection Agency, Washington, DC. EPA/540/R/99/005.

Wang, B., Lin, C., Zhang, X., Xu, D., Cheng, H., Wang, Q., Liu, X., Ma, J., 2018. Effects of geography, age, and gender on Chinese children's soil ingestion rate. Human and Ecological Risk Assessment: An International Journal 24,(7), 1983-1989. https://doi.org/10.1080/10807039.2018.1435255
